# Flow Cytometry for Diagnosing Sézary Syndrome When Skin Biopsies Are Inconclusive: A Case Report

**DOI:** 10.7759/cureus.107213

**Published:** 2026-04-17

**Authors:** Abdelhafid El Mrahi, Souha Denna, Mohammed Bensalah, Zerrouki Nassiba, Nada Zizi

**Affiliations:** 1 Dermatology, Mohammed VI University Hospital, Medical School of Oujda, Mohammed First University, Oujda, MAR; 2 Biological Hematology, Mohammed VI University Hospital, Medical School of Oujda, Mohammed First University, Oujda, MAR; 3 Epidemiology, Mohammed VI University Hospital, Medical School of Oujda, Mohammed First University, Oujda, MAR

**Keywords:** cutaneous t-cell lymphoma, diagnostic, flow cytometry, sézary syndrome, tnmb, trbc1

## Abstract

Sézary syndrome (SS) is a rare leukemic variant of cutaneous T-cell lymphomas, accounting for a small proportion of cases, estimated at less than 5% of all cutaneous T-cell lymphomas, characterized by pruritic erythroderma, lymphadenopathy, and circulating clonal T lymphocytes. Diagnosis can be challenging when skin biopsies are non-diagnostic, potentially delaying treatment. Peripheral blood flow cytometry (FC) is essential for detecting aberrant T-cells, assessing clonality, which may be supported in selected cases by T-cell receptor beta-chain constant region 1 (TRBC1) expression analysis, quantifying tumor burden, and guiding tumor-node-metastasis-blood (TNMB) staging and follow-up.

A 58-year-old man presented with diffuse pruritic erythroderma and lesions refractory to topical corticosteroids, systemic corticosteroids, methotrexate, and emollients. Repeated skin biopsies showed spongiotic or psoriasiform changes without evidence of malignancy. Peripheral blood smear revealed 5-7% Sézary cells. FC identified an aberrant cluster of differentiation (CD)4+ T-cell population representing 90% of lymphocytes, with CD7+, CD26−, and 97% TRBC1 positivity, confirming a clonal malignant population. Lymph node biopsy was consistent with T-cell lymphoma. TNMB classification was T4N3M0B2 (stage IVA2). The patient received peginterferon alfa-2a with topical corticosteroids and supportive care, leading to clinical and laboratory improvement. This case underscores the critical role of FC when skin biopsies are inconclusive. Comprehensive immunophenotyping, including TRBC1, confirms monoclonality, informs TNMB staging, and guides targeted therapy. Quantitative assessment of circulating malignant cells allows dynamic monitoring of treatment response. Early integration of peripheral blood FC in suspected SS improves diagnostic accuracy, reduces delays, and enables rapid initiation of targeted therapy, ultimately optimizing patient outcomes.

## Introduction

Sézary syndrome (SS) is a rare leukemic form of cutaneous T-cell lymphoma, characterized by generalized pruritic erythroderma, lymphadenopathy, and circulating malignant T lymphocytes known as Sézary cells [1,2]. Erythroderma is defined as erythema and scaling involving ≥80% of the body surface area and represents a key clinical feature and staging criterion in SS. Although uncommon, prognosis is poor, with a median survival of two to five years depending on disease stage and comorbidities [1,3].

Diagnosis can be challenging when skin biopsies are non-specific, often showing spongiotic or psoriasiform changes that mimic chronic eczema or psoriasis, which may delay therapeutic management [2,4,5]. Peripheral blood flow cytometry (FC) has become an essential tool in SS, enabling the detection of clonal T lymphocytes, detailed immunophenotypic characterization [cluster of differentiation (CD)4+, CD7-/+, CD26−, T-cell receptor beta-chain constant region 1 (TRBC1) clonality], The tumor-node-metastasis-blood (TNMB) classification proposed by the International Society for Cutaneous Lymphomas (ISCL)/ European Organization for Research and Treatment of Cancer (EORTC) is a standardized staging system for cutaneous T-cell lymphomas that assesses disease involvement in four compartments: skin (T), lymph nodes (N), visceral organs (M), and blood (B), and is essential for staging, prognosis, and treatment decisions [4,6-8].

This report illustrates a case in which FC was decisive for diagnosis despite non-contributive skin biopsies, highlighting its key role in the evaluation of erythrodermic patients.

## Case presentation

**Figure 1:**
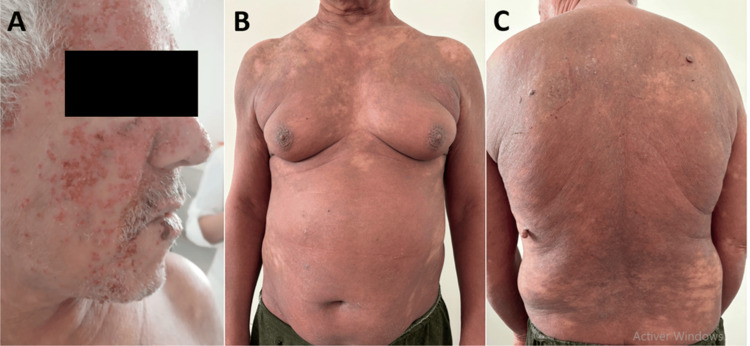
Clinical presentation of Sézary syndrome. (A) Infiltrated facial appearance with erosive lesions. (B) Anterior trunk showing diffuse erythema. (C) Posterior trunk showing extensive erythroderma.

A 58-year-old man with a history of schizophrenia and former smoking presented with severe pruritic erythroderma that had been evolving for one year. Initial lesions were erythematous and scaly, localized on the trunk, and were refractory to topical corticosteroids, systemic corticosteroids, methotrexate, and emollients. The patient reported associated asthenia without fever or weight loss. Clinical examination revealed diffuse erythroderma covering 92% of the body surface area, an infiltrated facial appearance with erosive lesions (Figure 1), and plantar keratoderma.

**Figure 2:**
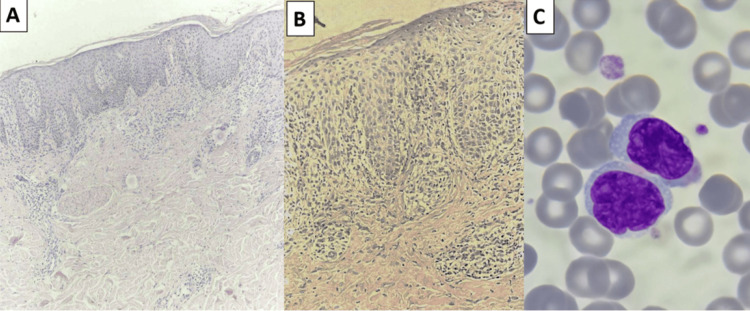
Histopathological and hematological findings. (A) H&E (×10): Acanthosis with compact parakeratotic hyperkeratosis, elongation of rete ridges, and a dermal polymorphic inflammatory infiltrate. (B) H&E (×20): Marked elongation of rete ridges with exocytosis of inflammatory cells and pigment incontinence. (C) Peripheral blood smear (May-Grünwald-Giemsa, ×1000) showing Sézary cells with atypical cerebriform nuclei and condensed chromatin.

**Figure 3:**
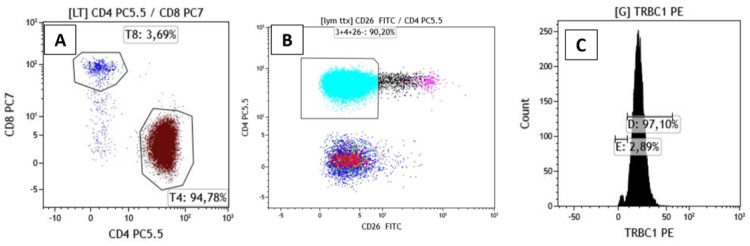
Flow cytometry findings of peripheral blood T-cell population. (A) CD4⁺ T-cell population representing 94% of lymphocytes with a CD4/CD8 ratio of 21. (B) Aberrant immunophenotype with insignificant loss of CD7and loss of CD26 expression. (C) TRBC1 expression in 97% of CD4⁺ T-cells, consistent with a clonal T-cell. population.

Multiple skin biopsies performed over six months were non-diagnostic, showing either spongiotic dermatitis or psoriasiform changes without evidence of malignancy (Figures 2A, 2B). Differential diagnoses included erythrodermic psoriasis, drug-induced erythroderma, and atopic dermatitis; however, clinical progression and hematological findings favored SS. Peripheral blood smear revealed 5-7% Sézary cells (Figure 2C). FC identified an aberrant CD4+ T-cell population representing 94% of lymphocytes, with a CD4/CD8 ratio of 21. The neoplastic cells showed insignificant loss of CD7 and loss of CD26 expression, and demonstrated TRBC1 positivity in 97% of CD4+ T-cells, consistent with a clonal malignant T-cell population. This population accounted for approximately 90% of total lymphocytes, corresponding to an absolute count of 5000 cells/µL (Figure 3).

**Figure 4:**
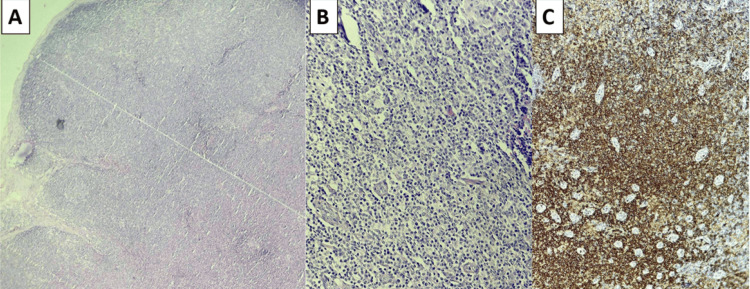
Histopathological and immunohistochemical findings of lymph node biopsy. (A) H&E (×4): Low-power view showing complete effacement of nodal architecture by a diffuse lymphoid proliferation. (B) H&E (×20): Higher magnification showing medium-sized atypical lymphoid cells with irregular nuclear contours and hyperchromatic to vesicular chromatin. (C) Immunohistochemistry showing strong CD4 expression in lymphoid cells.

Lymph node biopsy demonstrated infiltration consistent with T-cell lymphoma and Complete effacement of nodal architecture (Figure 4).

The patient presented with erythroderma involving more than 80% of the body surface area, consistent with the T4 stage. Peripheral blood involvement with circulating atypical lymphocytes supported B2 classification. Lymph node assessment revealed complete effacement by lymphoma. Based on these findings, the disease was staged as T4N3M0B2 according to the TNMB (ISCL/EORTC) classification, corresponding to stage IVA2.

**Figure 5:**
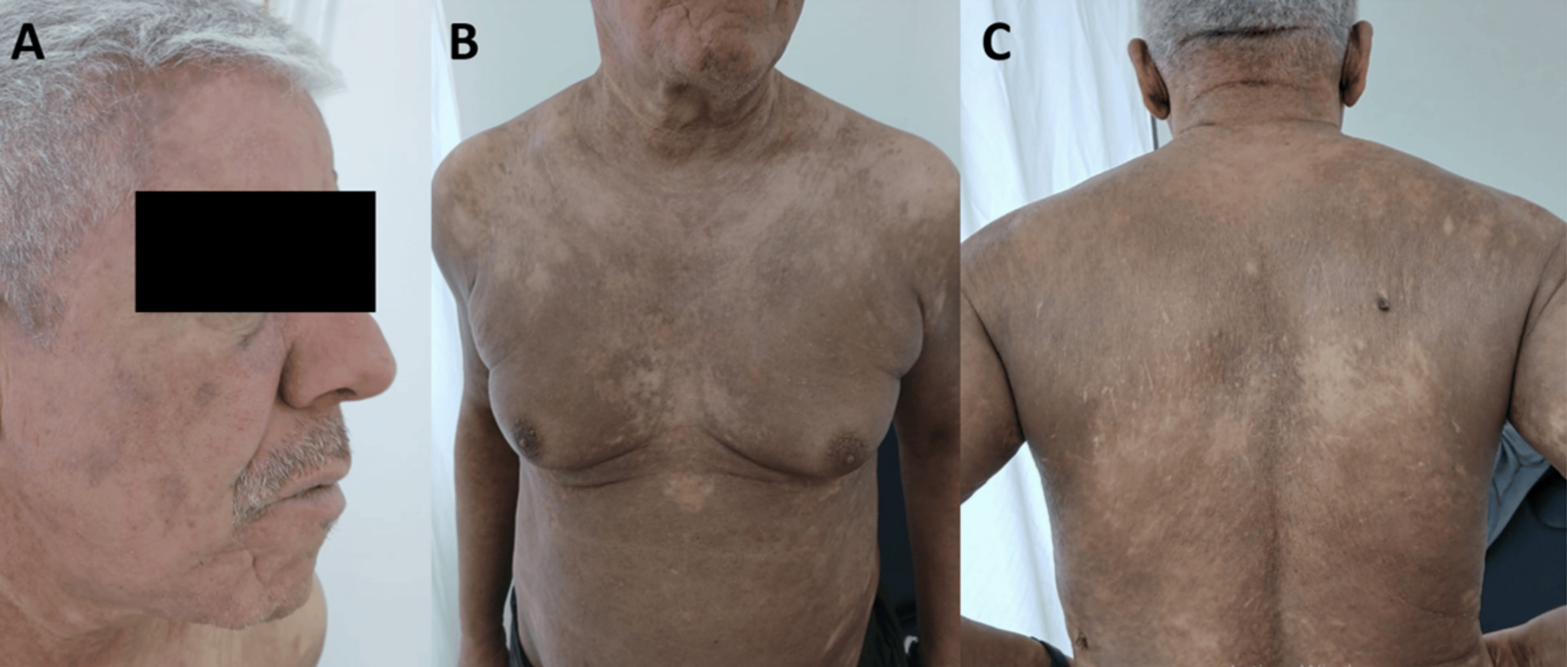
Clinical improvement following two months of treatment. (A) Resolution of facial infiltration and erosive lesions. (B,C) Resolution of truncal erythema with residual post-inflammatory hyperpigmentation.

Therapeutic management was initiated after referral to the hematology-oncology department and consisted of pegylated interferon alfa-2a (180 µg/week) combined with topical corticosteroids and supportive care measures. Extracorporeal photopheresis (ECP), a recommended modality in advanced stages, was not accessible in our setting. Nevertheless, the patient exhibited marked clinical improvement, accompanied by a reduction in circulating aberrant T-cell burden on follow-up evaluation. (Figure 5).

## Discussion

SS often presents a significant diagnostic challenge, as its clinical manifestations overlap with those of chronic inflammatory dermatoses, and skin biopsy results are frequently nonspecific. In this patient, repeated biopsies revealed only spongiotic or psoriasiform changes without evidence of malignancy, which could have delayed diagnosis if based solely on histopathology [2,4,7-9]. This underscores the crucial role of peripheral blood FC, particularly when skin biopsies are non-contributive. It allows the identification of circulating CD4+ T lymphocytes with an aberrant immunophenotype, providing definitive evidence of malignancy even in the absence of histopathological confirmation.

Assessment of TRBC1 clonality was particularly important in this case. This marker is based on the mutually exclusive expression of the constant region of the T-cell receptor β-chain, enabling FC-based assessment of T-cell clonality. It therefore supports the identification of monoclonal T-cell populations, a fundamental criterion for distinguishing Sézary syndrome from reactive lymphocytoses and other benign conditions [4,6,8,10]. Compared with T-cell receptor (TCR) gene rearrangement studies, this approach provides a rapid and widely applicable surrogate marker of clonality, although molecular assays remain the reference standard in selected cases. Although loss of CD7 is a commonly reported feature of Sézary cells, it is not indispensable for diagnosis; identification of a clonal population with other aberrant markers, such as CD26−, may be sufficient to establish the diagnosis [4,6,8,9]. This highlights that comprehensive immunophenotypic profiling, rather than reliance on a single marker, is essential for accurate diagnosis.

FC also provides quantitative information regarding circulating malignant cell burden, which is critical for B staging in the TNMB system and prognostic assessment [6-9,11,12]. Monitoring the proportion of aberrant T lymphocytes over time enables clinicians to evaluate treatment response and detect early signs of relapse, allowing rapid adaptation of therapeutic strategies. In this patient, the observed decrease in circulating aberrant T-cells closely correlated with clinical improvement, demonstrating the value of FC not only for diagnosis but also for dynamic disease monitoring.

B2 blood involvement in the ISCL/EORTC TNMB classification is defined by a high tumor burden in peripheral blood, including ≥1000 Sézary cells/µL or an elevated CD4/CD8 ratio associated with a clonal T-cell population. In this case, the patient demonstrated approximately 5000 Sézary cells/µL, a markedly increased CD4/CD8 ratio, and a TRBC1-restricted CD4+ T-cell population, collectively supporting B2 classification [4,11].

Sézary syndrome carries an unfavorable prognosis, with a median overall survival of approximately two to four years. Outcomes are primarily determined by TNMB stage, particularly B2 blood involvement, along with patient age and overall disease burden.

The management of Sézary syndrome requires a multidisciplinary, individualized approach based on a risk- and stage-adapted strategy. It is mainly centered on non-cytotoxic systemic therapies, including bexarotene, interferon-α, and histone deacetylase (HDAC) inhibitors, as well as immunotherapeutic approaches such as ECP, a procedure in which collected leukocytes are treated with 8-methoxypsoralen and exposed to UVA light before reinfusion, inducing apoptosis of malignant lymphocytes and promoting an immune-mediated anti-tumor response [4,13]. This modality is commonly used as a first-line treatment in erythrodermic cutaneous T-cell lymphomas. Targeted monoclonal antibodies, including mogamulizumab and brentuximab vedotin, have demonstrated efficacy in selected patients. Conventional systemic chemotherapy is generally reserved for refractory or advanced disease due to its limited duration of response. Allogeneic hematopoietic stem cell transplantation remains the only potentially curative option in carefully selected patients.

Regarding treatment, peginterferon alfa-2a was selected based on disease stage, patient comorbidities, and the availability of therapies. While novel targeted agents such as mogamulizumab (anti-CCR4) or brentuximab vedotin (anti-CD30) have demonstrated efficacy in SS, they were not accessible in our clinical setting. Anti-tumor necrosis factor (TNF) therapies are not standard for SS and were therefore not used. Peginterferon alfa-2a remains a practical and effective option for reducing circulating Sézary cells and managing symptoms, in line with current guidelines [1,4,6,8].

Overall, this case emphasizes that FC is indispensable for establishing a diagnosis of Sézary syndrome, guiding TNMB staging, assessing prognosis, and monitoring therapeutic response, particularly when skin biopsies are inconclusive. Its early integration into the diagnostic workflow prevents misdiagnosis, reduces delays, and enables rapid initiation of targeted therapy, thereby improving patient outcomes [4,6-9,11,13]. The combination of clinical evaluation, histopathology, and detailed immunophenotypic profiling constitutes the optimal approach for accurate diagnosis and management of Sézary syndrome.

## Conclusions

SS represents a major diagnostic challenge, particularly when skin biopsies are non-contributive. Peripheral blood FC plays a central role in the diagnostic workup by enabling the detection of circulating clonal T-cell populations, quantification of tumor burden, support for TNMB staging, and longitudinal disease monitoring over time. Early integration of this technique in erythrodermic patients improves diagnostic accuracy and facilitates timely initiation of appropriate management strategies. This case illustrates the value of incorporating FC early in the diagnostic workup of Sézary syndrome, particularly when histopathology is inconclusive. However, these findings are based on a single case and should therefore be interpreted within this limited context.
